# Home for the Dying

**Published:** 1891-07-11

**Authors:** 


### Editor's Letter-Box.

[Our correspondents are reminded that prolixity is a great bar to publication, and that brevity of style and conciseness of statement greatly facilitate early insertion].

### HOME FOR THE DYING.

We, the undersigned, beg leave to bring before the public, through the medium of your columns, the great and pressing need that exists for a home for the reception of dying persons in London, and would call attention to the following facts :—

There are at all times amongst us many impoverished invalids, who are suffering from mortal disease such as advanced consumption, cancer, and the like, and whose end is not far off. Their helpless condition has deprived them of whatever means of support they may have possessed. These people are very often not strictly suitable for admission to general hospitals, for such institutions are rather intended for alleviation and cure of disease than to afford a home or shelter to the hopelessly dying. Some exceptions exist to this rule, for example, the cancer wards of the Middlesex Hospital; on the other hand, the several Homes for Incurables appear to be designed for chronic cases of long duration.

We are strongly impressed with the conviction that there is thus a great gap in the Christian charity of our metropolis, and that it only needs to be understood to be filled. The small efforts already being made in this direction fall certainly far short of reaching the need of the case, and the best proof that can be offered of this is seen in the fact that at "Friedenheim," a small but little-known private home for the dying, having only ten beds, which has been open for five years past, about 300 applications from cases of the kind described above have been received annually, only about forty of whom could be accepted.

At the present juncture it is proposed to raise a sum of £10,000, which would be adequate for the purchase and fitting up of a suitable home for fifty beds. About £1,900 have already been guaranteed from private sources.

It is impossible in a short letter to enter into details, but all information will be gladly supplied by the Hon. Secretary, "Friedenheim," 133, Mildmay Road, London, N.

As the Council of this Home we earnestly commend its enlargement to the attention of the Christian public.

| | |
|---|---|
| R. Phayre, K.C.B. (Chairman.) | John Langton, F.R.C.S. |
| J. H. Tritton (Hon. Treasurer). | Hugh M. Matheson. |
| Thomas Barlow, M.D. | J. E. Mathieson. |
| C. Y. Biss, M.D. | J. L. Maxwell, M.D. |
| W. Mitchell Carruthers. | J. F. Morton, Colonel. |
| T. A. Denny. | H. Sinclair Patterson, M.D. |
| John C. Fraser. | |
| A. Pearce Gould, M.S. | A. T. Schofield, M.D. |
| T. Henry Green, M.D. | Nevile Sherbrooke. |
| S. H. Habershon, M.D. | H. W. Webb-Peploe. |

